# Do people with HIV infection have a normal life expectancy in the era of combination antiretroviral therapy?

**DOI:** 10.1186/1741-7015-11-251

**Published:** 2013-11-27

**Authors:** Caroline A Sabin

**Affiliations:** 1 Research Department of Infection and Population Health, UCL, Royal Free Campus, Rowland Hill Street, London NW3 2PF, UK

**Keywords:** Human immunodeficiency virus, Combination antiretroviral therapy, Life expectancy, Trends, Mortality

## Abstract

There is evidence that the life expectancy (LE) of individuals infected with the human immunodeficiency virus (HIV) has increased since the introduction of combination antiretroviral therapy (cART). However, mortality rates in recent years in HIV-positive individuals appear to have remained higher than would be expected based on rates seen in the general population. A low CD4 count, whether due to late HIV diagnosis, late initiation of cART, or incomplete adherence to cART, remains the dominant predictor of LE, and thus the individual’s disease stage at initiation of cART (or thereafter) certainly contributes to these higher mortality rates. However, individuals with HIV also tend to exhibit lifestyles and behaviors that place them at increased risk of mortality, particularly from non-AIDS causes. Thus, although mortality rates among the HIV population may indeed remain slightly higher than those seen in the general population, they may be no higher than those seen in a more appropriately matched control group. Thus, further improvements in LE may now only be possible if some of the other underlying issues (for example, modification of lifestyle or behavioral factors) are tackled.

## Introduction

Approximately 34.3 million people worldwide are thought to be infected with the human immunodeficiency virus (HIV) [1]. Left untreated, HIV is inevitably fatal, with a median survival time from seroconversion of 8 to 10 years [2]. However, the widespread introduction of combination antiretroviral therapy (cART) in many countries in the mid-1990s resulted in a rapid and dramatic reduction in mortality in those living with HIV [3,4]. Although the early cART regimens often included drugs with side effects that limited their efficacy, the drugs used as part of modern cART combinations are generally easier to take, have fewer side effects, and are more forgiving of minor lapses in adherence. As a result, immunological and virological responses to cART have continued to improve over time, with resulting continued reductions in mortality [5,6]. HIV has now come to be viewed by many as a chronic disease and, for the first time, the HIV research community has started to discuss the possibility that life expectancy (LE) in those infected with HIV may now be approaching that seen in the general population.

The aim of this review is to describe changes in LE in the HIV-positive population since the introduction of cART, and to consider whether this has now reached the same level as in those without HIV infection.

### What is life expectancy?

LE is an important indicator of health that is used widely by governments, healthcare agencies, and insurance companies to monitor trends in survival over time, and to determine resource allocation [7]. Formally, LE indicates the average number of years that a person would be expected to survive beyond a given age. That given age would usually be birth [8]; however, in the context of HIV, the given age may be difficult to interpret as, in most cases, individuals are not born with HIV but acquire it at some point during their life. Thus, LE is commonly quoted from a specific given age [9-11] or after some specific event, such as HIV diagnosis [12,13]. Of note, LE at a particular age is not the same as LE at birth minus that age, as LE at a particular age is calculated after conditioning on the fact that the individual has already survived to that age.

To describe the effect of a particular infection, such as HIV, on LE, investigators may prefer to report the potential years of life lost due to that infection. These may be ‘productive’ life years lost before the age of 65 years [9], or may be overall years of life lost [14]. Alternatively, investigators may report the potential gains in LE that could be achieved if that infection (in this case, HIV) were to be eliminated from the population [15-17], the excess mortality rates due to HIV [18], or the standardized mortality ratio (SMR) or mortality rate ratio [19,20], both of which provide a relative measure of the mortality rate in HIV-positive individuals compared with the expected mortality rate in an age-matched uninfected population. The variety of statistics that may be quoted, and the different ages at which LE may be expressed, complicates attempts to summarize LE in the cART era. Table 1 lists reported estimates of LE in the cART era from resource-rich settings, which range from 19.9 years at the age of 25 years in Denmark [21], to around 75 years from birth in the UK [8].

**Table 1:** Estimates of LE reported in the cART era

**Reference**	**Cohort/study name**	**Country of study**	**LE in HIV-positive population**	**LE in general population**
Nakagawa *et al*. [8]	Computer simulation (HIV Synthesis)	UK	LE at birth: 75.0 years if diagnosed with HIV with high CD4 count; 71.5 years if diagnosed with HIV with low CD4 count	LE at birth: estimated from model to be 82.0 years if not infected with HIV
The Antiretroviral Therapy Cohort Collaboration [9]	ART-CC	Multi-country study (Europe and North America)	LE at age 20: 43.1 years. LE at age 35: 31.7 years	Not stated
Johnson *et al*. [10]	IeDEA-SA	South Africa	LE at age 20: 27.6 years in men; 36.8 years in women. LE at age 60: 10.1 years in men; 14.4 years in women	Not stated
Mills *et al*. [11]	The AIDS Support Organization (TASO) cohort	Uganda	LE at age 20: 26.7 years. LE at age 35 years: 27.9 years	LE at age 20: 41 years
Losina *et al.*[12]	Computer simulation (CEPAC)	USA	LE at age 33: 22.66 years if optimally diagnosed and treated; 19.36 years if treated with cART and adherence follows normal patterns	LE at age 33: 42.91 years for general population; 34.58 years if risk profile similar to those with HIV
Bor *et al*. [17]		KwaZulu-Natal, South Africa	No specific estimates	LE at birth: 52.3 years in 2000; 49.2 years in 2003; 60.5 years in 2011
Lohse *et al*. [21]	Danish HIV Cohort Study	Denmark	LE at age 25: 8 years in 1995 to 1996; 23 years in 1997 to 1999; 33 years in 2000 to 2005	LE at age 25: 51 years
May *et al*. [23]	UK Collaborative HIV Cohort Study	UK	LE at age 20: 39.5 years in men; 50.2 years in women. LE at age 35: 30.1 years in men; 37.7 years in women	LE at age 20: 57.8 years in men; 61.6 years in women. LE at age 35: 43.5 years in men; 46.9 years in women
van Sighem *et al*. [41]	ATHENA Cohort	The Netherlands	LE at age 25: 52.7 years in men; 57.8 years in women	LE at age 25: 53.1 years in men; 58.1 years in women

*Abbreviations*: cART, combination antiretroviral therapy; LE, life expectancy.

### Changes in LE in the cART era

It is clear that LE has increased since the introduction of cART. Using data from the large CASCADE Collaboration, Bhaskaran [18] found a continued narrowing in the gap in mortality rates between those seen in individuals infected with HIV with known dates of HIV seroconversion and those that would have been expected based on a demographically similar HIV-negative population. Excess mortality rates in the HIV-positive population dropped by 94% from 31.4 per 1000 person-years (PYRS) prior to 1996 to 6.1 per 1000 PYRS in 2004 to 2006. Mortality rates among 43,355 cART-naive participants in the Antiretroviral Therapy Cohort Collaboration (ART-CC) dropped similarly from 16.3 per 1000 PYRS in 1996 to 1999, to 10.0 per 1000 PYRS in 2003 to 2005 [9]. LE at 20 and 35 years increased from 36.1 and 25.0 years to 49.4 and 37.3 years, respectively, over the same period, with the potential years of life lost decreasing from 366 per 1000 PYRS to 189 per 1000 PYRS. Among participants with acquired immune deficiency syndrome (AIDS) in the Longitudinal Study of Ocular Complications in AIDS [22], excess mortality decreased by 8.0% per year from the period 1999 to 2001 to the period 2006 to 2007. LE at age 25 years in the Danish HIV cohort increased from only 8 years in the pre-cART era (1995 to 1996) to 33 years in 2000 to 2005, with LE for a similarly aged uninfected Danish person during that period being 51 years [21]. Among individuals starting cART in the UK Collaborative HIV Cohort (CHIC) Study, LE at 20 years increased from 30.0 years if cART was started during 1996 to 1999 to 45.8 years if cART was started during 2006 to 2008 [23]. Of note, improvements in LE in the cART era are not restricted to resource-rich settings: the overall population LE at birth in KwaZulu-Natal, South Africa, is reported to have increased from 49.2 years in 2003 (prior to the scale-up of antiretroviral therapy), to 60.5 years in 2011 [17].

### Predictors of LE in the cART era: the role of disease stage

Despite the dramatic improvements in LE witnessed since the introduction of cART, LE may still not have reached the levels seen in the uninfected population. Bhaskaran [18] reported that even by 2003 to 2005, excess mortality rates in the CASCADE Collaboration remained elevated at 6.1 per 1000 PYRS, and in the ART-CC, potential years of life lost remained high (189 per 1000 PYRS) over the period 2003 to 2005 [9]. LE in patients starting cART in 2008 in the UK CHIC Study remained lower than that seen in the UK general population (59 years at age 20) [23]. Among women in the US Women’s Interagency HIV Study (WIHS), the SMR dropped from 24.7 in 1996 to a plateau of 10.3 during 2001 to 2003, despite the addition of a group of younger and healthier women into the cohort in 2001 to 2002 [19].

The disease stage of individuals at the time of initiation of cART, and shortly thereafter, may at least partly contribute to the higher than expected mortality rates seen in recent years (Table 2). In the Longitudinal Study of Ocular Complications in AIDS [22], excess death rates ranged from 128 per 1000 PYRS in individuals who had cytomegalovirus retinitis, a viral load of greater than 400 copies/ml, and a CD4 count of less than 200 cells/mm^3^, to only 8 per 1000 PYRS for individuals lacking these factors. Interestingly, although excess mortality rates in this study dropped in the cART era by 8.3% per year in those with a CD4 count of less that 200 cells/mm^3^, no significant reduction was seen in those with higher CD4 cell counts. In ART-CC participants [24], the lowest SMR was seen in men who have sex with men (MSM), who did not have AIDS at cART initiation and who had attained a viral load of 500 copies/ml or lower and a CD4 cell count of 350 cells/mm^3^ or higher by 6 months after starting cART. By contrast, the highest SMR was seen in injection drug users who failed to attain a suppressed viral load by 6 months and in whom the CD4 cell count remained at less than 50 cells/mm^3^.

**Table 2:** Summary of factors that may influence LE in people with HIV infection

**Sociodemographic factors**	**Lifestyle/behavioral factors**	**HIV-related factors**
Gender	Smoking	Late HIV diagnosis
Age	Alcohol use	CD4 count at cART initiation
Co-morbidities related to aging	Recreational and injection drug use	CD4 count and HIV RNA attained on cART
Ethnic group/country of origin	Viral hepatitis co-infection	Clinical AIDS prior to cART initiation
Place of residence/neighborhood	Sexually transmitted infections	Attendance at outpatient clinics
Socioeconomic status		Adherence to cART

*Abbreviations*: cART, combination antiretroviral therapy; LE, life expectancy.

The important association between the pre-cART CD4 count and LE has been described in several other studies. In the UK CHIC Study [23], individuals started on cART in line with UK guidelines (at a CD4 cell count of 200 to 350 cells/mm^3^) experienced a LE at age 20 of 53.4 years, only marginally shorter than that seen in the general male (57.8 years) and female (61.6 years) populations. By contrast, LEs at age 20 were only 41.0 and 37.9 years among those started on cART at a CD4 count of 100 to 199 and less than 100 cells/mm^3^, respectively. Among cART-treated South African individuals, LE at age 20 ranged from 43.1 years if the CD4 count was 200 cells/mm^3^ or higher to 29.5 years if the CD4 count was 50 cells/mm^3^ or lower [10]. In Australian cART-treated individuals [25], the SMR increased from 1.5 among individuals with a CD4 count of 500 cells/mm^3^ or higher to 8.6 among those with a CD4 cell count of 350 cells/mm^3^ or lower. Finally, among HIV-positive individuals in the Study of Fat Redistribution and Metabolic change in HIV Infection (FRAM), mortality rates were 2.3 times higher than in HIV-negative controls in individuals with a CD4 count of greater than 350 cells/mm^3^, but 6.3 times higher in those with a CD4 count of less than 350 cells/mm^3^[26]. Thus, it is clear that a low CD4 count, whether due to late diagnosis of HIV, late initiation of cART, or incomplete adherence to cART, remains the dominant predictor of LE in the cART era.

### Predictors of LE in the cART era: the role of non-HIV factors

Although stage of HIV disease at cART initiation is strongly associated with LE, other factors may also play a role. (Table 2) Individuals with HIV are known to exhibit lifestyles and behaviors that put them at higher risk of mortality than the general population, regardless of HIV status, including higher rates of smoking, alcohol and recreational drug use, and viral and sexually transmitted co-infections [27-30]. Current smoking was an additional risk factor for death in HIV-positive individuals in the FRAM Study [26], and in a recent study from the Danish HIV Cohort, Helleberg *et al*. [31] reported that those with HIV may now lose more life years to smoking than to HIV itself. Among participants in the ART-CC [9], injection drug users had a LE that was around 13 years shorter at age 20, and 10 years shorter at age 35, than non-injection drug users. The percentage of participants in this study with a SMR less than 2 (that is, individuals whose mortality patterns most closely resembled those in the general population) was 46% in MSM, 42% in those infected with HIV through heterosexual sex, and 0% among injection drug users; the corresponding percentages of participants with a SMR greater than 10 (individuals with the worst mortality patterns) were 4%, 14%, and 47%,s respectively [24], confirming the negative impact of injection drug use and/or hepatitis co-infection on overall mortality rates [19,21].

Although these non-HIV factors may have only a limited influence on deaths from AIDS-related causes, they may play a more major role in deaths from non-AIDS causes, which appear to have increased in frequency in the cART era. In the WIHS Study [19], deaths from non-AIDS causes increased in the cART era, and by 2001 to 2004, they accounted for the majority of deaths that occurred; it was this increase in non-AIDS deaths that was thought to contribute to the plateau in the SMR seen from 2001 among women in the study. Whereas the mortality rate ratio for deaths from non-AIDS causes in non-injection drug users in the Danish HIV Cohort had dropped from 4.5 in 1995 to 1.3 in 2008, it had increased from 7.0 to 10.3 over the same period in injection drug users [32]. In a direct comparison with the Multicenter AIDS Cohort Study (MACS), Wada [33] reported that median LE for non-AIDS causes was almost 10 years shorter in women in the WIHS (55.9 years) than in men in the MACS (66.0 years), contributing to an overall difference in age at death between men and women of 11.6 years. Further evidence of the potential role of non-HIV factors in mortality rates comes from Alabama [34], where patients who missed visits in the first year after initiating outpatient treatment for HIV had over twice the rate of long-term mortality compared with those attending all scheduled appointments, and from Canada, where a three-fold increased risk of death was seen in cART-treated HIV-positive individuals who lived in neighborhoods with a high concentration of injection drug users, relative to those who lived in neighborhoods with a high concentration of MSM [35].

To investigate the potential effect of these external factors on the mortality rates seen, Lohse [36] used data from the Danish general population to show that only around 55% of deaths that occurred in the Danish HIV cohort could be attributed to HIV, with 32% of deaths being attributed to hepatitis C virus co-infection and/or other co-morbidities, and the remaining 14% being unrelated to either HIV or co-morbidities. Losina and colleagues [12] used the CEPAC model, a state-transition model of HIV infection, to quantify the potential influence on LE of various lifestyle and behavioral factors. They found that in the general US population, LE at age 33 (the mean age at seroconversion in the USA) was around 43 years [12], but this dropped to 34.58 years when the authors selected a cohort from the HIV-negative population that matched their HIV-positive population in terms of several lifestyle and sexual risk factors. The authors were then able to estimate that HIV infection, when appropriately treated and diagnosed at an early stage, would lead to a further loss of LE of around 11.92 years, with late diagnosis, late initiation of cART, and early discontinuation of cART further reducing LE by an additional 3.3 years [12].

### Can we improve LE further?

Late HIV diagnosis remains extremely common in many countries [37], and has been reported to be a major risk factor for mortality [38]. In Brazil, it was estimated that 95.5% of deaths occurring in the first year after diagnosis were attributable to late diagnosis [39]; study investigators estimated that averting late diagnosis would have reduced the AIDS mortality rate 2003 to 2006 by 39.5%, a similar reduction to that produced by cART. In the UK, earlier diagnosis would have reduced short-term (first year after diagnosis) mortality by 84% in MSM [38] and by 56% in those infected heterosexually [40]. Using the HIV Synthesis model, a stochastic computer simulation model of HIV progression, Nakagawa [8] showed that LE from birth was 71.5 years, with 10.5 years lost to HIV infection, in a scenario in which diagnosis occurred at a late stage of HIV infection (median CD4 count 140 cells/mm^3^), but under a scenario of earlier diagnosis (median CD4 count 432 cells/mm^3^), LE from birth was 75.0 years, with only 7.0 years lost, on average, due to HIV. Thus, earlier diagnosis of HIV might go some way to improve LE further.

Among those diagnosed and receiving cART, efforts to ensure that all individuals attain optimal CD4 levels may also lead to improvements in LE. Lewden calculated SMR for individuals in the COHERE collaboration who had attained a CD4 count of 500 cells/mm^3^ or higher on cART [20]. For men, attaining a CD4 count of 500 cells/mm^3^ or higher for just over 1 year was sufficient to ensure that their mortality rates were similar to those in the general population. For women, however, SMR remained above 1, even among those who had maintained a CD4 count of 500 cells/mm^3^ or higher for over 5 years. The potential for further improvement in LE was also studied in the Dutch ATHENA cohort [41]; LE at age 25 among HIV-positive participants who had been diagnosed during 1998 to 2007 and who remained AIDS-free and untreated for 24 weeks after diagnosis was 52.7 years in men (versus 53.1 years in the general population) and 57.8 years in women (versus 58.1 years). The authors noted that individuals included in the study were highly selected (injection drug users were excluded) with a median CD4 count at 24 weeks after diagnosis of 480 cells/mm^3^, and therefore the outcomes reported reflect the potential outcomes that might be feasible in a group of patients diagnosed and treated at an early stage of infection. Of note, there is some evidence to suggest a small potential benefit of cART (through a reduction in CD4 loss) if it is initiated during primary HIV infection [42]. Although such benefits may translate into further improvements in LE, any effect at a population level is likely to be small, given the difficulties in diagnosing individuals with HIV infection at such an early stage.

Earlier HIV diagnosis and optimal cART initiation aside, do we still have some way to go to improve LE, or have we already reached the maximum LE that might be anticipated in this population? Although LE in those with HIV infection is generally compared with that seen in the general population in the same country, LEs vary tremendously both between and within countries. In the UK, for example, male LE at birth in 2007 to 2009 ranged from 84.4 years for those living in parts of London to 73.1 years for those living in parts of Glasgow [43]. Even within a city such as London, there may be large differences in LE in different areas, as shown by the Lives on the Line project (http://life.mappinglondon.co.uk/). These differences may be explained by differences in the characteristics of those living in different regions, particularly socioeconomic status, lifestyle factors, and dietary factors. When LE is compared between the HIV-positive and the general populations, therefore, the two populations may have a different underlying risk of mortality, and LEs may be expected to differ from that in the general population. The identification of appropriately matched HIV-negative control populations, with similar lifestyle and behavioral characteristics, for the provision of comparative estimates of LE, would go some way to addressing this concern.

This inability to eliminate residual confounding is a limitation of any comparison based on observational data. However, LE also suffers from several other limitations. Firstly, LE is generally based on current mortality rates and does not take into consideration any improvements to patient management that may occur in the future (leading to an underestimate of future LE) nor to any longer-term possible adverse outcomes of cART or HIV infection (leading to an overestimate of future LE). Secondly, the estimation of LE often requires long-term extrapolation of mortality rates from individuals followed over a relatively short period. After all, HIV has only been around for 30 years or so, a relatively short time compared with the length of an individual’s lifetime. Finally, LE is only as good as the ascertainment of deaths within a cohort; where deaths are not fully ascertained, LE may appear artificially high. Using information collected from cohort studies in West Africa, Cote d’Ivoire, and Burkina Faso, Lewden *et al*. [44] reported that the highest estimates of mortality were seen in cohorts with the lowest rates of loss to follow-up. Verguet *et al*. [45] subsequently reported that whereas the best estimate of life years gained by a person in Africa in the first 5 years after starting cART was 2.1 [45], this estimate could drop by approximately 14% if mortality rates among those lost to follow-up were assumed to be 100%, or could increase by 19% if zero mortality was assumed in this group. In cohorts participating in the ART-CC, incomplete death ascertainment was reported to contribute to the higher mortality rates seen in the North American compared with European cohorts, although other patient factors also played a role [46].

## Conclusions

With the limitations described above in mind, it is possible that LE may now have reached levels that we would expect to see in this population. At this stage, it is possible that further major improvements in LE may only be achievable by tackling some of the other underlying issues, such as earlier HIV diagnosis (through enhanced opportunities for testing and greater awareness of the early signs of HIV infection) and improved retention in HIV care, earlier cART initiation, or the modification of lifestyle or behavioral factors.

## Abbreviations

AIDS: Acquired immune deficiency syndrome; ART-CC: Antiretroviral therapy cohort collaboration; cART: Combination antiretroviral therapy; HIV: Human immunodeficiency virus; LE: Life expectancy; MACS: Multicenter AIDS cohort study; MSM: Men who have sex with men; PYRS: Person-years; SMR: Standardised mortality ratio; UK CHIC study: UK collaborative HIV cohort study; US: United States; WIHS: Women’s interagency HIV study.

## Competing interests

The author declares that she has no competing interests.

## Author’s information

CS is a professor of Medical Statistics and Epidemiology at University College London (UCL). She has worked for many years on the analysis of large observational HIV databases, with a particular interest in raising awareness of the biases inherent in these databases. She is the principal investigator on the UK CHIC Study, principal statistician on the D:A:D Study, and has worked with many other research groups in the UK and elsewhere.
